# The silent struggle: assessing anxiety prevalence in Marrakech’s hypertensive population

**DOI:** 10.1186/s12889-025-22459-z

**Published:** 2025-03-28

**Authors:** Fatima Zahra Boukhari, Safae Belayachi, Firdaous Essayagh, Ahmed Anouar Naji, Hajar Lemriss, Khaoula Addakiri, Meriem Essayagh, Sanah Essayagh, Touria Essayagh

**Affiliations:** 1 Faculté des Sciences et Techniques, Laboratoire Agroalimentaire et Santé, Hassan First University of Settat, Settat, Morocco; 2 Faculté des Sciences Juridiques, Économiques et Sociales, Laboratoire Droit Privé et Enjeux de Développement, Université Sidi Mohamed Ben Abdellah, Fès, Morocco; 3 Institut Supérieur des Sciences de la Santé, Laboratoire Sciences et Ingénierie Biomédicale, Biophysique et Santé, Hassan First University of Settat, Settat, Morocco; 4 Office National de Sécurité Sanitaire des Produits Alimentaires, Oriental, Morocco

**Keywords:** Self-reported anxiety, Hypertension, Stress, Pain and physical discomfort, Social support, Diet

## Abstract

**Background:**

Anxiety is a disorder that may negatively impact the quality of life for people with hypertension. In Marrakech, epidemiological data on anxiety in hypertension patients are scarce, preventing holistic treatment for hypertensive patients and favoring the development of comorbidities. This study aims to assess the prevalence of self-reported anxiety among hypertensive patients receiving care in primary healthcare centers in Marrakech.

**Methods:**

Between May 2021 and December 2022, a cross-sectional study of 1053 hypertensive patients who visited primary healthcare centers in Marrakech was carried out. Socio-demographic information, behavioral and clinical information, hypertensive treatment characteristics, and the care-patient-physician triad were all collected via a face-to-face questionnaire. The Generalized Anxiety Disorder Scale (GAD-7) was used to assess self-reported anxiety. Multivariate logistic regression was used to identify risk factors for self-reported anxiety.

**Results:**

A total of 348 (33.1%) of those with hypertension overall reported having anxiety symptoms. 301 (86.5%) patients were females, and the mean age was 61.5 ± 9.6 years (mean ± standard deviation). High stress, moderate stress, family history of hypertension, pain and physical discomfort, non-purchase of drug if it is out of stock at the primary healthcare centers, living in urban areas, lack of social support, and non-consumption of five fruits and vegetables per day were identified as risk factors of self-reported anxiety.

**Conclusion:**

Self-reported anxiety is prevalent in one third of hypertensive patients in Marrakech. Hence, greater emphasis should be placed on the mental health component when managing hypertensive patients in primary healthcare centers.

**Supplementary Information:**

The online version contains supplementary material available at 10.1186/s12889-025-22459-z.

## Introduction

Hypertension is a public health issue and a risk factor for non-communicable illnesses afflicting 1.28 billion people worldwide. 82.0% of them reside in low- and middle-income countries [1]. Each year, hypertension causes nine million premature deaths in the world [2]. In Morocco, the prevalence of non-communicable illnesses has grown as a result of demographic change, urbanization, and a sedentary lifestyle. According to the most current stepwise research on risk factors for noncommunicable diseases, which included 5429 persons aged 18 and older, the prevalence of hypertension was 29.3% in 2018 [3]. The Ministry of Health and Social Protection is implementing a program to prevent and control hypertension since 1996 in an effort to lower premature mortality, morbidity, and incapacitating conditions caused by hypertension. Over 1.28 million hypertensive individuals received treatment in 2021, while a total of 89,786 people received a diagnosis at primary healthcare centers [4].

Mental disorders are also a public health problem, with a prevalence of 7.3% worldwide [5]. In Morocco, this prevalence was 9% [6]. Anxiety is defined by the World Health Organization (WHO) as the feeling of imminent and indeterminate danger accompanied by uneasiness, agitation, helplessness, or even annihilation [7, 8]. Several studies have reported a positive association between high blood pressure and anxiety. Indeed, in 2015, an association between hypertension and self-reported anxiety was described in a systematic review of 13 studies [9, 10]. Demographic characteristics such as age [11], sex [12], environmental characteristics such as exposure to traffic noise [13], and behavioral characteristics such as sedentary lifestyle, smoking, and alcohol abuse were the main risk factors associated with comorbid hypertension and anxiety [14, 15].

Failure to screen for anxiety in hypertensive patients can have negative impacts on their treatment. It can promote non-adherence to antihypertensive drugs, to dietary rules, and to medical monitoring, and even lead to uncontrolled blood pressure and complications [16]. Anxiety can even impair a patient’s quality of life [17], and increase healthcare costs [18].

To better direct public policies for the prevention and treatment of hypertension, knowledge of the prevalence of anxiety in hypertensive patients and its risk factors is crucial. Therefore, the aim of our study was to determine the prevalence of self-reported anxiety among hypertensive patients monitored at Marrakech’s primary healthcare centers.

## Methods

We carried out cross-sectional research between the 26th of May 2021 and the 30th December of 2022. The study included hypertensive patients monitored at primary healthcare centers. We used a two-stage sampling method. The primary unit was made up of primary healthcare centers. The secondary unit consisted of hypertensive patient aged 18 years or older who had been on pharmacological therapy for hypertension for at least six months, were monitored at primary healthcare centers in Marrakech, and provided their consent to participate in the study. Pregnant women were not included in the study.

### Sample size and data collection tool

We determined the sample size based on a 50% estimated prevalence of self-reported anxiety, a 95% confidence range, a 5% margin of error, a 20% non-response rate, and a cluster effect of two. The required patient number was 922.

We used a standardized questionnaire administered face-to-face to gather information on sociodemographic and economic factors, behavioral and clinical traits, antihypertensive treatment characteristics, and the relationship between patients, the healthcare system, and the doctor.

The socio-demographic and economic data included: age in years, sex (female or male), area of residence (urban or rural), marital status (single or partnered), education (can read and write or can neither read nor write), occupation (with or without), monthly income (less than 150 dollars, between 150 and 199 dollars, between 200 and 299 dollars, between 300 and 499 dollars, and more than or equal to 500 dollars), and health insurance (yes or no).

### Operational definition

Self-reported anxiety assessment: the Generalized Anxiety Disorders-7 (GAD-7) was used to assess self-reported anxiety in hypertensive patients. The GAD-7 consists of seven questions to which patients respond by “never”, “several days”, “more than half the days” and “almost every day”. The following questions were asked: Over the last two weeks, how often have you been bothered by the following problems: (i) Feeling nervous, anxious, or on edge?; (ii) Unable to stop or control worrying?; (iii) Worrying excessively about several things?; (iv) Having trouble relaxing?; (v) Being so restless that it is hard to sit still?; (vi) Getting quickly irritated or upset?; (vii) Fearing that something terrible could occur?. Each question was scored on a scale of zero to three. zero for “never”, one for “several days”, two for “more than half the days” and three for “almost every day”. The sub-scores of the seven questions were put together to yield a total score that ranged from 0 to 21. The following is how the score was interpreted: A score of 0 to 4 indicated no symptoms; a score of 5 to 9 indicated mild symptoms; a score of 10 to 14 indicated moderate symptoms; and a score of 15 to 21 indicated severe symptoms. People who received a score of 5 or higher were deemed to have self-reported anxiety [19, 20].

Blood pressure: An electronic sphygmomanometer with an adjustable cuff (Micro Life Pro M with a 3-mmHg accuracy) was used for measuring blood pressure. We referred to the European Society of Arterial Hypertension and the European Society of Cardiology (ESH/ESC) guidelines for blood pressure regulation to identify hypertensive patients [21].

We utilized a digital scale (with an accuracy of 100 g) to measure the body weight of barefoot patients in little clothes. We used a measuring rod to determine the height. The patients stood, barefoot, feet together, arms at their sides, at attention [22]. Overweight and obesity were determined using WHO guidelines [23].

We determined the existence of social support by asking participants if they received social support.

We reported physical pain or discomfort by asking participants if they had chest pain, lower back pain, headaches, or any other signs that produced pain or discomfort.

We reported the presence of reduced mobility by asking participants if they had difficulty going around on foot.

We reported a lack of autonomy by asking participants if they had difficulty taking care of themselves (like if they could dress or wash themselves).

We assessed the healthcare system’s relationship with patients by asking the following questions: (i) How far is it from your home to the primary healthcare center?; (ii) How long does it take you to go to the primary healthcare center?; (iii) Which kind of transport do you use when going to the primary healthcare center? If the distance between the participants’ home and the primary healthcare center is greater than or equal to 6 km and the time required to get to the primary healthcare center is greater than or equal to 30 min, and the participants need to go via a mode of transport, we reported unsatisfactory relationship with the healthcare system.

### Statistical analysis

All data were imported into Excel and analyzed using Epi-Info-7. To describe continuous variables, the mean and standard deviation were used. For categorical variables, we used numbers and percentages. The Pearson chi-square test or, where applicable, Fisher’s exact test were used to compare categorical variables. The analysis of variance (ANOVA) or Mann-Whitney tests were used to compare continuous variables, if appropriate. In the multiple logistic regression, we included all variables with a p-value less than 0.20 in the bivariate analysis. The adjusted odds ratio (AOR) and its 95% confidence interval were used to assess the association between the risk factor and the presence of self-reported anxiety.

## Results

### Demographic and socioeconomic characteristics

The demographic and socio-economic characteristics of hypertensive patients are summarized in Table 1. A total of 1053 patients were approached, with a prevalence of 33.1% self-reported anxiety. The average age of the patients was 63,0 ± 09.9 years; 811 (77.0%) were female; and 300 (28.5%) had a monthly family income of less than $150.

**Table 1:** Demographic and socio-economic characteristics of hypertensive patients followed in primary healthcare centers, Marrakech, 2021–2022

	Total participants *n* (%)	Self-reported anxiety *n* (%)	No self-reported anxiety *n* (%)
Total participants	1053 (100)	348 (33.1)	705 (66.9)
Means age ± SD (in years)	63.0 ± 09.9	61.5 ± 09.6	63.7 ± 10.0
Sex			
Female	811 (77.0)	301 (86.5)	510 (72.3)
Male	242 (23.0)	47 (13.5)	195 (27.7)
Area of residence			
Urban	788 (74.8)	290 (83.3)	498 (70.6)
Rural	265 (25.2)	58 (16.7)	207 (29.4)
Age group (in years)			
≥80	58 (05.5)	14 (04.0)	44 (06.2)
[70–79]	213 (20.2)	54 (15.5)	159 (22.6)
[60–69]	439 (41.7)	146 (42.0)	293 (41.6)
[50–59]	248 (23.6)	95 (27.3)	153 (21.7)
[40–49]	85 (08.1)	36 (10.3)	49 (06.9)
[29–39]	10 (00.9)	3 (00.9)	07 (01.0)
Marital status			
†Single	379 (36.0)	123 (35.3)	256 (36.3)
‡Partnered	674 (64.0)	225 (64.7)	449 (63.7)
Education			
Can neither read nor write	743 (70.6)	253 (72.7)	490 (69.5)
Can read and write	310 (29.4)	95 (27.3)	215 (30.5)
Occupation			
Without	962 (91.4)	316 (90.8)	646 (91.6)
With	91 (08.6)	32 (09.2)	59 (08.4)
Monthly income ($)			
<150	300 (28.5)	118 (33.9)	182 (25.8)
150–199	428 (40.6)	133 (38.2)	295 (41.9)
200–299	269 (25.6)	82 (23.6)	187 (26.5)
300–499	35 (03.3)	1 3 (03.7)	22 (03.1)
≥500	21 (02.0)	02 (0.6)	19 (02.7)
Health insurance			
No	168 (16.0)	61 (17.5)	107 (15.2)
Yes	885 (84.0)	287 (82.5)	598 (84.8)

SD: standard deviation

†Single refers to being single, divorced, or widowed

‡Partnered means married

### Behavioral characteristics

Table 2 summarizes those 101 hypertensive patients (09.6%) were tobacco users, 47 (04.5%) were alcohol consumers, 937 (89.0%) had stress, 746 (70.8%) had insufficient physical activity, 669 (63.5%) were sedentary, 567 (53.8%) did not eat five fruits and vegetables per day, and 367 (34.8%) stated that they did not benefit from social support.

**Table 2:** Behavioral characteristics of hypertensive patients followed at primary healthcare centers, Marrakech, 2021–2022

	Total participants *n* (%)	Self-reported anxiety *n* = 348	No self-reported anxiety *n* = 705
General knowledge of hypertension			
Unsatisfactory	1032 (98.0)	341 (98.0)	691 (98.0)
Satisfactory	21 (02.0)	07 (02.0)	14 (02.0)
Tobacco consumption			
Yes	101 (09.6)	21 (06.0)	80 (11.3)
No	952 (90.4)	327 (94.0)	625 (88.7)
Alcohol consumption			
Yes	47 (04.5)	11 (03.1)	36 (05.11)
No	1006 (95.5)	337 (96.8)	669 (94.9)
Stress			
Intense	267 (25.4)	175 (50.3)	92 (13.0)
Moderate	670 (63.6)	164 (47.1)	506 (71.8)
Low	116 (11.0)	09 (02.6)	107 (15.2)
Physical activity			
Unsatisfactory	746 (70.8)	271 (77.9)	475 (67.4)
Satisfactory	307 (29.2)	77 (22.1)	230 (32.6)
Sedentary			
Yes	669 (63.5)	235 (67.5)	434 (61.6)
No	384 (36.5)	113 (32.5)	271 (38.4)
Salt diet			
Low-salt	284 (27.0)	99 (28.5)	185 (26.2)
Salty	769 (73.0)	249 (71.5)	520 (73.8)
Consumption of five fruits and vegetables			
No	567 (53.8)	213 (61.2)	354 (50.2)
Yes	486 (46.2)	135 (38.8)	351 (49.8)
Difficulty following the diet			
Yes	259 (24.6)	91 (26.1)	168 (23.8)
No	794 (75.4)	257 (73.9)	537 (76.2)
Social support			
No	367 (34.8)	180 (51.7)	187 (26.5)
Yes	686 (65.2)	168 (48.3)	518 (73.5)

### Clinical characteristics

According to Tables 3 and 508 (48.2%) of hypertensive patients had a comorbidity, 458 (43.5%) had diabetes, 499 (47.4%) had a duration of hypertension greater than or equal to five years, 601 (57.1%) had a family history of hypertension, 79 (07.5%) had reduced autonomy, 587 (55.8%) experienced pain and physical discomfort, and 843 (80.1%) were overweight or obese.

**Table 3:** Clinical characteristics of hypertensive patients followed at primary healthcare centers, Marrakech, 2021–2022

	Total participants *n* (%)	Self-reported anxiety *n* = 348	No self-reported anxiety *n* = 705
Comorbidity			
Yes	508 (48.2)	186 (53.5)	322 (45.7)
No	545 (51.8)	162 (46.5)	383 (54.3)
Diabetes			
Yes	458 (43.5)	169 (48.6)	289 (41.0)
No	595 (56.5)	179 (51.4)	416 (59.0)
Dyslipidemia			
Yes	56 (05.3)	22 (06.3)	34 (04.8)
No	997 (94.7)	326 (93.7)	671 (95.2)
Duration of hypertension			
More than five years	499 (47.4)	181 (52.0)	318 (45.1)
Less than or equal to five years	554 (52.6)	167 (48.0)	387 (54.9)
Family history of hypertension			
Yes	601 (57.1)	248 (71.3)	353 (50.0)
No	452 (42.9)	100 (28.7)	352 (50.0)
Controlled blood pressure			
No	749 (71.1)	256 (73.6)	493 (69.9)
Yes	304 (28.9)	92 (26.4)	212 (30.1)
Autonomy			
Yes	79 (07.5)	37 (10.6)	42 (06.0)
No	974 (92.5)	311 (89.4)	663 (94.0)
Reduced mobility			
Yes	126 (12.0)	64 (18.4)	62 (08.8)
No	927 (88.0)	284 (81.6)	643 (91.2)
Pain and physical discomfort			
Yes	587 (55.8)	240 (69.0)	347 (49.2)
No	466 (44.2)	108 (31.0)	358 (50.8)
Overweight or obesity			
Yes	843 (80.1)	296 (85.1)	547 (77.6)
No	210 (19.9)	52 (14.9)	158 (22.4)

### Characteristics of the care-patient-doctor system and antihypertensive drug

Amongst 1053 participants, 873 (82.9%) had a bad relationship with the healthcare system, 507 (48.1%) didn’t think their doctor was willing to pay attention to their questions about their sickness, 637 (60.5%) reported antihypertensive drug non-availability at the primary healthcare center, 263 (25.0%) reported adverse effects from the antihypertensive drug, and 252 (23.9%) reported not buying antihypertensive drug in case of stockout at the primary healthcare centers “Table 4”.

**Table 4:** Characteristics of the care-patient-doctor system and antihypertensive drug in hypertensive patients followed at primary healthcare centers, Marrakech, 2021–2022

	Total participants *n* (%)	Self-reported anxiety *n* = 348	No self-reported anxiety *n* = 705
Patient-care system relationship			
Unsatisfactory	873 (82.9)	314 (90.2)	559 (79.3)
Satisfactory	180 (17.1)	34 (09.8)	146 (20.7)
Patient-doctor relationship			
Unsatisfactory	866 (82.2)	293 (84.2)	573 (81.3)
Satisfactory	187 (17.8)	55 (15.8)	132 (18.7)
Feel the doctor’s willingness to understand the patient’s hypertension concerns			
No	507 (48.1)	201 (57.8)	306 (43.4)
Yes	546 (51.9)	147 (42 2)	399 (56.6)
Availability of the drug			
No	637 (60.5)	258 (74.1)	379 (53.8)
Yes	416 (39.5)	90 (25.9)	326 (46.2)
Thinking you have a lot of drugs to take			
Yes	40 (03.8)	22 (06.3)	18 (02.5)
No	1013 (96.2)	326 (93.7)	687 (97.5)
Adverse drug effects			
Yes	263 (25.0)	107 (30.7)	156 (22.1)
No	790 (75.0)	241 (69.3)	549 (77.9)
Non-purchase of a drug if it is out of stock			
Yes	252 (23.9)	133 (38.2)	119 (16.9)
No	801 (76.1)	215 (61.8)	586 (83.1)
Self-monitoring			
No	849 (80.6)	294 (84.5)	555 (78.7)
Yes	204 (19.4)	54 (15.5)	150 (21.3)

### Self-reported anxiety

Self-reported anxiety was reported by 348 (33.1%) participants. Table 1 shows that they were 61.5 ± 9.6 years old on average, 301 (86.5%) were females, and 290 (83.3%) resided in cities. According to an examination of behavioral and clinical data, 339 (32.2%) were stressed, and 180 (51.7%) stated that they had not benefited from social support “Table 2”. A total of 248 (71.3%) had a family history of hypertension, 240 (69.0%) had pain or physical discomfort “Table 3”, and 133 (38.2%) did not buy drug if there was a supply deficit in primary healthcare centers “Table 4”. Ten (0.9%) individuals experienced severe anxiety, 65 (06.2%) mild anxiety, 273 (25.9%) light anxiety, and 705 (67.0%) had no anxiety symptoms.

During the bivariate analysis, the p-value was set at 0.20. As mentioned in “Table 5”, after bivariate analysis, 26 factors were associated with the presence of self-reported anxiety: (i) female sex; (ii) age; (iii) urban area; (iv) low monthly income; (v) tobacco consumption; (vi) alcohol consumption; (vii) stress; (viii) sedentary; (ix) non-consumption of five fruits and vegetables per day; (x) lack of self-monitoring of hypertension; (xi) lack of social support; (xii) presence of comorbidity; (xiii) presence of diabetes; (ivx) duration of hypertension greater than five years; (xv) family history of hypertension; (xvi) lack of autonomy; (xvii) pain and physical discomfort; (xviii) reduced mobility; (ixx) overweight or obesity; (xx) unsatisfactory relationship between the patient and the healthcare system; (xxi) patients’ perception of their doctor’s lack of willingness to understand their concerns regarding their hypertension; (xxii) availability of the drug; (xxiii) non-purchase of drug if it is out of stock at the primary healthcare centers; (ivxx) perception by the patient of having too much drug to take; (xxv) perception that the drug has more adverse effects than benefits; and (xxvi) unsatisfactory physical activity “Table 5”.

**Table 5:** Multivariate analysis (odds ratio, p-value) of risk factors associated with self-reported anxiety among hypertensive patients, Marrakech, Morocco, 2021–2022

	Bivariate analysis		Multivariate analysis	
	**COR (95% CI)**	*p-value*	**AOR (95% CI)**	*p-value*
Female sex	2.4 [1.72–3.47]	< 0.0001	1.5 [0.90–2.68]	0.10
Age in years		0.01		
≥80	0.7 [0.16–3.26]	0.69	1.1 [0.16–8.02]	0.88
[70–79]	0.8 [0.19–3.17]	0.74	0.8 [0.14–5.35]	0.88
[60–69]	1.2 [0.29–4.56]	0.82	1.2 [0.20–7.28]	0.81
[50–59]	1.4 [0.36–5.73]	0.59	1.2 [0.20–7.28]	0.81
[40–49]	1.7 [0.41–7.08]	0.45	1.6 [0.26–10.1]	0.60
[29–39]	1			
Urban area	2.1 [1.50–2.87]	< 0.0001	1.8 [1.11–2.87]	0.01
Monthly income ($)		0.009		
≤149	6.1 [1.40–26.8]	0.01	2.5 [0.52–12.8]	0.24
150–199	4.3 [0.98–18.5]	0.05	2.9 [0.59–14.2]	0.18
200–299	4.1 [0.94–18.2]	0.05	3.3 [0.68–16.3]	0.13
300–499	5.6 [1.12-28.0]	0.03	3.5 [0.61–20.7]	0.15
≥500	1			
Tobacco consumption	0.5 [0.30–082]	0.004	0.9 [0.39–2.46]	0.97
Alcohol consumption	0.6 [0.30–1.20]	0.13	1.0 [0.34–2.94]	0.98
Stress		< 0.0001		
Intense stress	22.6 [10.9–46.7]	< 0.0001	12.4 [5.6–27.2]	< 0.0001
Moderate stress	3.8 [1.90–7.78]	0.0002	2.4 [1.12–5.12]	0.02
Low stress	1			
Unsatisfactory physical activity	1.7 [1.26–2.29]	0.0003	1.1 [0.79–1.79]	0.38
Sedentarity	1.2 [0.99–1.70]	0.05	1.0 [0.34–2.94]	0.98
Not consuming five fruits and vegetables a day	1.5 [1.20–2.03]	0.0007	1.4 [1.01–1.97]	0.04
Lack of monitoring hypertension	1.4 [1.04–2.07]	0.02	1.2 [0.81–1.89]	0.31
Lack of social support	2.9 [2.26–3.88]	< 0.0001	1.6 [1.17–2.35]	0.004
Presence of comorbidity	1.3 [1.05–1.76]	0.01	0.8 [0.37–1.72]	0.57
Presence of diabetes	1.3 [1.04–1.75]	0.02	1.3 [0.62–2.85]	0.45
Duration of hypertension more than five years	1.3 [1.01–1.70]	0.03	0.9 [0.71–1.36]	0.95
Family history of hypertension	2.4 [1.87–3.25]	< 0.0001	2.2 [1.58–3.13]	< 0.0001
Lack of autonomy	1.8 [1.18–2.98]	0.008	1.4 [0.66–2.98]	0.37
Pain and physical discomfort	2.2 [1.74-3.00]	< 0.0001	1.8 [1.41–2.48]	< 0.0001
Reduced mobility	2.3 [1.60–3.40]	< 0.0001	1.4 [0.79–2.77]	0.21
Overweigh and obesity	1.6 [1.16–2.31]	0.003	1.0 [0.65–1.57]	0.95
Unsatisfactory patient relationship with the healthcare system	2.4 [1.62–3.59]	< 0.0001	1.2 [0.72–2.25]	0.39
Feel the doctor’s willingness to understand the patient’s hypertension concerns	1.8 [1.37–2.31]	< 0.0001	1.1 [0.81–1.68]	0.40
Availability of the drug	2.4 [1.86–3.26]	< 0.0001	1.2 [0.82-2.00]	0.26
Non-purchase of a drug if it is out of stock	3.0 [2.27–4.08]	< 0.0001	1.8 [1.27–2.76]	0.001
Thinking you have a lot of drugs to take	2.5 [1.36–4.86]	0.003	1.4 [0.64–3.25]	0.37
Adverse drug effects	1.5 [1.17–2.08]	0.002	1.1 [0.76–1.63]	0.56

COR Crude odds ratio, AOR Adjusted odds ratio, CI Confidence interval

### Multivariate analysis

After controlling for other variables, we identified the following factors to be associated with self-reported anxiety in hypertensive patients: (i) high stress compared to low stress (Adjusted Odd Ratio of 12.4; CI [5.66–27.2]); (ii) moderate stress compared to low stress (AOR of 2.4; CI [1.12–5.12]); (iii) family history of hypertension (AOR of 2.2; CI [1.58–3.13]); (iv) pain and physical discomfort (AOR of 1.8; CI [1.41–2.48]); (v) non-purchase of drug if it is out of stock at the primary healthcare centers (AOR of 1.8; CI [1.27–2.76]); (vi) urban area (AOR of 1.8; CI [1.11–2.87]); (vii) lack of social support (AOR of 1.6; CI [1.17–2.35]); and (viii) non-consumption of five fruits and vegetables per day (AOR of 1.4; CI [1.01–1.97]) “Table 5”.

## Discussion

To our knowledge, this is the first study in Morocco assessing the prevalence of self-reported anxiety in patients with hypertension followed at primary healthcare centers. Previous surveys carried out by our research team had dealt with uncontrol of high blood pressure and non-adherence to antihypertensive treatment without focusing on mental problems [24–26]. The national survey conducted in 2005 among the general population had shown a prevalence of self-reported anxiety of 9% [6]. In this study, among hypertensive patients, the frequency of self-reported anxiety was 33.1%, whereas it was 55.3% in a sample of 152 hypertensive patients in Brazil [27], 32.7% in a sample of 471 in Ethiopia [28], 23.1% in a sample of 322 in Nigeria [29], 21.7% in Russia, in a sample of 2775 hypertensive patients [30], 12.3% in China, in a sample of 4993 hypertensive patients [31], and 11.6% in Zhejiang in a sample of 891 hypertensive patients [9]. These disparities in frequency between countries might be attributed to the sample size and the heterogeneity of the techniques employed to assess self-reported anxiety. Hormonal differences between the sexes, psychosocial factors associated with feminine ideas of gender, and how parents interact with their baby at birth, depending on whether he is a boy or a girl, all influence how adults perceive their anxiety symptoms, putting women at a higher risk than men [32, 33]. Female sex was not associated with self-reported anxiety in our study.

Pogosova et al. link stress to self-reported anxiety; a similar finding was found in our study [30].

Low monthly household income, recurrent stock-outs of antihypertensive drugs at primary healthcare centers, financial difficulties in purchasing these drugs from pharmacies, complications from hypertension, and a lack of social support could explain this association. Moreover, the unsatisfactory relationship between the patient, the healthcare system, and the nursing staff is also contributing to the anxiogenic symptoms.

In this study, having a family history of hypertension was linked to self-reported anxiety. This might be explained by the illness’s potential consequences, negative familial experiences with hypertension, and the patient’s refusal to recognize the diagnosis.

In our study, living in an urban area was associated with self-reported anxiety. The 2023 study, done in the United Kingdom with 156,075 participants, found a significant association between self-reported anxiety and living in an urban area [34]. This could be associated with the high population density, pollution and noise, and the quick pace of life. It could also be explained by the social isolation that comes with anonymity in big cities.

Pain and physical discomfort in hypertensives could occur and lead to feelings of worthlessness and helplessness, low self-esteem, and increased in negative thoughts related to fears from complications from hypertension. Fear of pain during physical movement can lead to avoidance behavior that makes hypertensive patients more susceptible to anxiety. In our study, pain and physical discomfort were associated with self-reported anxiety.

Low monthly income per household, high cost of antihypertensive drugs, food inflation following the COVID-19 pandemic, the need to meet the basic needs of all family members at the expense of one’s own drug needs, and fear of developing complications are all elements that may explain the relationship between self-reported anxiety and non-purchase of antihypertensive drugs when out of stock in primary healthcare settings.

According to Strep de Vries et al., the presence of greenery causes a decrease in anxiety and a rise in feelings of well-being. It would result in a reduction in mental tiredness and a greater ability to recuperate from stress [35]. Indeed, interaction with nature stimulates people to engage in healthy activities such as physical activity and reduces the prevalence of worry. According to the scientific literature, vegetation has a psychological influence on human health. This evolutionary hypothesis supports the notion that the human species’ two million years of development in the natural environment would have left biological and genetic marks. And that any visual contact with the natural world, whether it be plants or water, will elicit a sense of security and well-being. The paucity of green space in metropolitan areas might explain the link between self-reported anxiety and the urban living environment in our study [36].

People’s mental health is influenced by social support through the effects it has on their emotions, cognition, and actions. As a result, the social network can influence a person’s behaviors and daily activities, such as sports and eating habits, as well as the development of positive psychological states such as a sense of belonging, security, stability, and a recognition of one’s value. The social network may also provide important information to stimulate the adoption of healthy behaviors as well as physical or financial assistance to increase overall well-being. Integration into a social network is important for all of these reasons because it lessens feelings of despondency [37], increases motivation to take care of oneself and enhances immunological functions while increasing endocrine system processes. In our research, a lack of social support was linked to self-reported anxiety.

Because of their vitamin and mineral content, vegetables, fruits, and plant ingredients may be anxiolytics [38], as may be other formulations containing a diverse spectrum of micronutrients [39]. These micronutrients, which include zinc and selenium, function as coenzymes in the production and regulation of neurotransmitters and neurotrophic factors [40–42] which may explain their role in mental well-being. Non-consumption of five fruits and vegetables per day was linked to self-reported anxiety in hypertensive patients in our study.

Our study has some limitations, such as social desirability bias encountered in collecting data on cigarette and alcohol consumption, as well as intentional lying bias in collecting data on monthly income. The patient’s declarations served as the basis for measuring social support. Future study should consider using more robust and accurate measurement methods, such as the Cohen and Wills social support scale.

Our study seems to have a selection bias; however, the female population’s predominance could be explained by the nature of the health programs given in primary health care facilities, which are primarily concerned with maternity and child health. It could also be explained by the fact that women prioritize their health more than males.

## Conclusion

Anxiety symptoms are present in hypertensive patients, which might affect the blood pressure reading and accordingly their management care and their quality of life. Risk factors such as stress, a family history of hypertension, physical pain and discomfort, not purchasing antihypertensive medication when it was out of stock at primary healthcare centers, a lack of social support, and not eating five fruits and vegetables per day were associated with the presence of self-reported anxiety.

Treating physicians should assess the presence of these risk factors, and regularly screen hypertensive patients for anxiety throughout their medical follow-up in order to ensure holistic medical care and be able to reduce complications linked to hypertension and improve the quality of life of hypertensive patients. Other similar studies should be carried out in other regions to confirm our results and be able to establish a causal link.

## Electronic supplementary material

Below is the link to the electronic supplementary material.

Supplementary Material 1: S1 English version of the Questionnaire. Used in face-to-face patient’s interview.

## Data Availability

All data generated or analyzed during this study are included in this published article.
