# Supplementary material for: The silent struggle: assessing anxiety prevalence in Marrakech’s hypertensive population
Source: BMC Public Health. 2025 Mar 28;25:1179. doi: 10.1186/s12889-025-22459-z (PMC11951773; doi:10.1186/s12889-025-22459-z)
Supplement: Supplementary file 1 — Supplementary Material 1: S1 English version of the Questionnaire. Used in face-to-face patient’s interview. [file 12889_2025_22459_MOESM1_ESM.pdf]

Questionnaire of hypertensive patients

Survey Information

Survey location: .... Date of survey: /__//__/ /__/__/ /__//__/
Day Month Year

Interviewer ID /____//____/ (Initial of interviewer + initials of primary care health center)

Time of the interview: /__//__/ /__//__/ Hour Minute

Participant ID Number

/__//__/ /____//____/ (patient order number + interviewer ID)

Consent form has been read to the patient : /__/ yes /__/ no

If not, read it to the patient

Consent has been obtained, orally or written form: /__/ yes /__/ non

If no, end the interview

The information collected through this document will be treated with the utmost confidentiality and will be subject to the rules of ethics relating to the respect of patient privacy and medical confidentiality.

Demographic Information

Gender: Male /__/ Female /__/ **Age:** /___/ years **or** date of birth /__//__//__/__/

Residence Area: Urban /__/ Rural /__/

Marital status: Married /__/ Divorced /__/ Widowed /__/ Single /__/

Education: Can read and write /__/ cannot read nor write /__/

Level of education: Illiterate /__/ Primary /__/ Secondary /__/ University /__/

Occupation: Without /__/ with /__/ if with, specify: ....

Monthly income per household in Dhs: ≤1500 /__/]1500-≤2000] /__/]2000- 3000] /__/]3000-4999] /__/ ≥5.000 /__/

Health insurance coverage: Mutual /__/ RAMED/__/ Insurance/__/ Without /__/

Risk factors

Blood pressure duration: ________ years

**General knowledge about hypertension**			
**Symptoms**	**Yes**	**No**	
Headache			
Auditory whistling			
Blurred vision (feeling of flies in front of the eyes)			
Dizziness			
Palpitation (fast heart rate)			
Difficulty breathing			

Epistaxis (bleeding nose)			
Hematuria (presence of blood in the urine)			
Œdema			
**Complications**	**Yes**	**No**	
Stroke			
Heart attack			
Kidney damage			
Eye damage			
**Prevention**	**Yes**	**No**	
Dietary and hygiene rules			
- loss of weight			
- Reduction in alcohol consumption			
- Reduction in tobacco consumption			
Physical activity			
Stress avoidance			
Dietary compliance			
Compliance with treatment			
Taking medication that can lead to an increase in hypertension			
Self-measurement of blood pressure and regular medical (monitoring) follow-up			
**Behaviors**	**Yes**	**No**	
Tobacco			If yes, specify :<br>/__/ Current smoker<br>/__/ Former smoker<br>How many years have you stopped smoking?: …………………………<br>Number of cigarettes per day:<br>……………………………………….
Alcohol			If yes, specify :<br>/__/ Current drinker<br>/__/ Former Drinker<br>For how many years :<br>………………………………………<br>Quantity of glasses per day:<br>……………………………………….

Sedentarity			How many hours the patient remains seated or lying down: ……………………………………….
Physical activity<br><br>- Physical activity of more than 10 minutes duration that has resulted in increased heart rate<br><br>- Physical activity of more than 10 minutes duration in a row **to move from one place to another**, resulting in increased heart rate<br><br>- Physical activity of more than 10 minutes **leisurely** that has led to an increase in heart rate (walking, running, fitness)			If yes, how many times per week and how many hour<br><br>If yes : /__/ Low /__/ Moderate /__/ Vigorous<br>………………………………………<br>If yes : /__/ Low /__/ Moderate /__/ Vigorous<br>………………………………………<br><br>If yes : /__/ Low /__/ Moderate /__/ Vigorous<br><br>………………………………………
NB: low refers to intensity if the activity is carried out only one day or during less than 2 hours a week, while intense if it will be carried out at least for 4 days / week or more than 7 hours /week; it will be called moderate for the rest.			
Stress			/__/ No /__/ Moderately /__/ A lot
**Dietary compliance**	**Yes**	**No**	
-Consumption of salt			If yes : /__/ Semi salty /__/ Salty
Consumption of vegetables per day			/__/ Zero vegetables /__/ one to 2 vegetables /__/ 3 to 4 vegetables /__/ 5 vegetables or more
Consumption of five fruits per day			/__/ Zero fruits /__/ one to 2 fruits /__/ 3 to 4 fruits /__/ 5 fruits or more
Difficulty following the diet			Why? ……………………………….
**Family history of hypertension**			If yes : /__/ Mother /__/ Father /__/ Sister /__/ Brother /__/ Grandparent
**Comorbidity**	**Yes**	**No**	
- Heart disease			If yes, type: …………………………………..
- Diabetes			If yes, type: /__/ type 1 /__/ type 2

- Dyslipidemia			
- Chronic renal failure			
**Complication**			/__/ Stroke /__/ Heart damage /__/ kidney damage
**Name of antihypertensive treatment**			………………………………………..
- Monotherapy	**Yes**	**No**	/__/ Angiotensin II converting enzyme inhibitor /__/ thiazide diuretic beta-blocker /__/ calcium channel blocker /__/ Angiotensin II receptor antagonist
- Dual therapy			/__/ Angiotensin II converting enzyme inhibitor /__/ thiazide diuretic beta-blocker /__/ calcium channel blocker /__/ Angiotensin II receptor antagonist
- Triple therapy			/__/ Angiotensin II converting enzyme inhibitor /__/ thiazide diuretic beta-blocker /__/ calcium channel blocker /__/ Angiotensin II receptor antagonist
Generic drug			
**Duration of last treatment time in months** : ………………………………………..			
**Adherence to treatment**			
Did you forget to take your medication this morning?			/__/ Yes /__/ No
Have you been out of medication since the last consultation?			/__/ Yes /__/ No. If yes : Did you: /__/ buy it from the pharmacy /__/ take another drug /__/ remained without /__/ other
Have you had to take your treatment late compared to the usual time?			/__/ Yes /__/ No

Have you ever missed your treatment because some days because of your memory lacking		/__/ Yes /__/ No
Have you ever missed treatment because some days you feel that your treatment is doing more harm than good		/__/ Yes /__/ No
Do you think you have too many pills to take?		/__/ Yes /__/ No
**Self-monitoring**	/__/ Yes /__/ No	
Do you measure your home blood pressure?	/__/ Yes /__/ No	If no, why :<br>/__/ No instrument of measure<br>/__/ Other to specify : …………..<br>……………………………………
**Relationship with the care system**		
- Do you go to a **doctor** for your blood pressure ?	/__/ Yes /__/ No	If yes, does it belong to the sector /__/ Public /__/ Private
- Frequency of **medical** appointments	/__/ Once a month /__/ Every 2 months /__/ Every 3 months /__/ Every 6 months /__/ Never /__/ Just if I fell myself bad	
- What is the distance between your house and health center	/__/ <6 km /__/ Between 6 and 10 km /__/ >10 Km	
- Time taken to reach the health center	/__/ Less than 30 min /__/ Between 30 min and 1h /__/ More than one hour	
- Transportation mode	/__/ Walk /__/ Chariot /__/ Taxi /__/ Bus /__/ Car /__/ Bicycle	
- Availability of the drug for blood pressure	/__/ Yes /__/ No	
**Patient doctor relationship**		
- How long does the doctor allow you during the consultation in term of time in minutes	…………………………………………………………	
Do you discuss with your doctor your personal concerns/issues about hypertension?	/__/ Yes /__/ No	
Do you feel your doctor's desire to understand you better ?	/__/ Yes /__/ No	
Do you have blood pressure goals set for you ?	/__/ Yes /__/ No	

Have you missed any follow-up appointments ?	/__/ Yes /__/ No. If yes, how many times: …………
Does someone call you when you miss your appointment ?	/__/ Yes /__/ No
**Do you attend an association for hypertensive patients ?**	/__/ Yes /__/ No
**Does your family support you in taking care of your hypertension ?**	/__/ Yes /__/ No
Over the last two weeks, how often have you been bothered by the following problems?	
Feeling nervous, anxious, or on edge ?	/__/ Never /__/ Several days /__/ More than half the days /__/ Almost every day
Unable to stop or control worrying?	/__/ Never /__/ Several days /__/ More than half the days /__/ Almost every day
Worrying excessively about several things?	/__/ Never /__/ Several days /__/ More than half the days /__/ Almost every day
Having trouble relaxing?	/__/ Never /__/ Several days /__/ More than half the days /__/ Almost every day
Being so restless that it is hard to sit still?	/__/ Never /__/ Several days /__/ More than half the days /__/ Almost every day
Getting quickly irritated or upset?	/__/ Never /__/ Several days /__/ More than half the days /__/ Almost every day
Fearing that something terrible could occur?	/__/ Never /__/ Several days /__/ More than half the days /__/ Almost every day

Mobility	/__/ I have no difficulty going around on foot /__/ I have difficulty going around on foot /__/ I am confined to bed
Autonomy	/__/ I have no problems taking care of myself /__/ I have some problems washing or dressing myself /__/ I am unable to wash or dress myself

Physical pain / Discomfort	/__/ I have no pain or discomfort /__/ I have moderate pain or discomfort /__/ I have extreme pain or discomfort

Anthropological measurement

	Systolic, mmHg	Diastolic, mmHg
1st measure, right arm		
1st measure, left arm		
2nd measurement at the high pressure arm		
3rd measurement at the high pressure arm		
**Mean**		

Anthropological measurement

Weight in Kg	
Height in cm	
Hip circumference in cm	
Waist circumference in cm	
Blood pressure during last three visits	……………., ……………., …………….

Follow-up examination

ECG: /__/ yes /__/ no Result:

Electrolytes or ions (sodium, potassium): /__/ yes /__/ no Result:

Creatinine blood test: /__/ yes /__/ no Result:

Fasting blood glucose or glycated Hemoglobin (HbA1c): /__/ yes /__/ no Result:

Lipid profil: /__/ yes /__/ no

Total Cholesterol: /__/ yes /__/ no Result:

C-HDL: /__/ yes /__/ no Result:

C-LDL: /__/ yes /__/ no Result:

Triglycerides: /__/ yes /__/ no Result:
